# Changes of potential shorty-chain fatty acids producing bacteria in the gut of patients with spinal cord injury: a systematic review and meta-analysis

**DOI:** 10.3389/fmicb.2025.1483794

**Published:** 2025-02-27

**Authors:** Zaowei Zhong, Fei Fan, Junqiao Lv, Zhiqiang Wang, Beiyang Wang, Chen Deng, Lin Sun

**Affiliations:** ^1^Third Hospital of Shanxi Medical University, Shanxi Bethune Hospital, Shanxi Academy of Medical Sciences, Tongji Shanxi Hospital, Taiyuan, China; ^2^Department of Orthopedics, The Third People’s Hospital of Datong, Datong, China

**Keywords:** spinal cord injury, trauma spinal cord injury, gut microbiota, meta-analysis, short-chain fatty acids

## Abstract

Gut bacteria that potential produce short-chain fatty acids (SCFAs) influences the recovery of motor function in the host in patients with spinal cord injury (SCI). We aimed to conduct a review and meta-analysis of the literature on gut microbiota in SCI patients. Following the Preferred Reporting Project for Systematic Review and Meta-Analysis (PRISMA), we searched Embase, PubMed, Cochrane Library, Web of Science (WOS) and ClinicalTrials.gov. The search period was from inception to March 31, 2024. We reported standardized mean differences (d) with 95% confidence intervals (CI) and used funnel plots and Egger tests to assess publication bias. The subacute of SCI data set revealed the microflora changes in the subacute phase, and meta-analysis summarized the changes in the chronic phase. Eleven studies (720 participants) were included, 2 phyla, 1 order, and 14 genus meta-analyses performed. No substantial heterogeneity was observed, and significant publication bias was not found among the studies included. In the subacute phase of spinal cord injury, the relative abundance of Bacteroidetes, Clostridiales, Faecalbacterium, Ruminococcus, Coprococcus, Lachnospira, Dorea, Prevotella, Roseburia, Atopobium, Bifidobacterium, Bacteroides, and Blautia increased. Firmicutes and Lactobacillus decreased. In the chronic phase, Firmicutes decreased in the SCI group. Bifidobacterium, Bacteroides, Blautia, and Eubacterium were found to have a higher average proportion of abundance in patients with SCI compared to non-SCI persons, and Clostridiales, Ruminococcus, Faecalbacterium, Coprococcus, and Lachnospira showed a lower relative abundance in SCI. The genus of potential SCFAs-producing bacteria is lower in the chronic phase of spinal cord injury than in the subacute phase, and gut dysbiosis is present in both the subacute and chronic phases.

## 1 Introduction

Spinal cord injury (SCI) is a severe trauma to the central nervous system, often accompanied by complications such as immune dysfunction, intestinal dysfunction, and autonomic dysfunction. Both SCI and its associated complications impose a significant burden on patients’ lives and contribute to a high social cost (Ahuja et al., 2017; Ding et al., 2022; GBD 2016 Traumatic Brain Injury and Spinal Cord Injury Collaborators, 2019). The gut microbiota is believed to play an important role in host digestion, production and absorption of nutrients, immune system, and other important physiological functions (Jandhyala et al., 2015). Due to the enormous potential of the gut microbiota, more and more experts and scholars are paying attention to the gut microbiota of patients. Targeting the gut microbiota of SCI patients may have therapeutic value (Jogia and Ruitenberg, 2020; Turroni et al., 2018).

In recent years, there has been evidence of gut dysbiosis in humans and mice after SCI (Bazzocchi et al., 2021; Kigerl et al., 2016; Yu et al., 2021). Some animal studies have shown that dysbiosis of the gut microbiota can exacerbate inflammation of the spinal cord and colon, impairing the recovery of motor function (Kigerl et al., 2016; Rong et al., 2021; Schmidt et al., 2021). After specific types of fecal transplantation or probiotic supplementation treatment, it was observed that SCI mice had better motor function recovery than the control group (He et al., 2022; Jing et al., 2021; Kigerl et al., 2016). These animal studies also found that intervention in the gut micro-biota of SCI mice resulted in an increase in the detection level of SCFA in feces. Animal experiments have been conducted to investigate the effects of SCFAs on SCI mice. Feeding SCFAs resulted in better motor function recovery, higher neuronal survival rate, and better axon formation than the control group (Jing et al., 2023; Jing et al., 2021). Intestinal symbiotic bacteria produce SCFAs in the colon through anaerobic fermentation. Short-chain fatty acids have many beneficial properties and can improve neurological function in various central nervous system diseases through immune, vagus, endocrine, or other humoral pathways (Dalile et al., 2019; Dicks, 2022; Khan et al., 2021; Li et al., 2021). SCFAs might significantly impact the recovery of motor function and other physiological functions in patients with SCI. We propose that potential SCFA-producing bacteria may serve as critical targets for enhancing motor function recovery through modulation of the gut microbiota in patients with spinal cord injury.

Therefore, the changes of potential SCFAs-producing bacteria after human spinal cord injury deserve our special attention. Differences in the relative abundance of bacteria at the genus level have been observed across studies following spinal cord injury, which may be attributed to variations in the study populations’ genetics, diet, geography, and analytical procedures. We analyzed the data of the subacute stage of spinal cord injury to obtain the changes in the abundance of SCFAs-producing bacteria in the subacute stage. We included the studies of the chronic stage of spinal cord injury for meta-analysis so as to objectively compare the changes in the relative abundance of intestinal SCFA bacteria in the subacute stage and the chronic stage of spinal cord injury. Our research is expected to provide theoretical support for future researchers to intervene in the development of spinal cord injury by targeting the gut microbiota of SCI, enriching the theory of the brain-gut axis.

## 2 Materials and methods

### 2.1 Design and registration

The study was reported according to the Preferred Reporting Items for Systematic Review and Meta-Analysis (PRISMA) (Page et al., 2021). The protocol was informed by the Cochrane Handbook for Systematic Review of Interventions and registered via the International Prospective Register of Systematic Reviews (PROSPERO) (CRD42023417200).^1^

### 2.2 Search strategy

We selected relevant studies published before March 31, 2024, by searching Embase, PubMed, Cochrane Library, Web of Science (WOS), and ClinicalTrials.gov. We applied no language restrictions. A combination of Mesh with free text search was applied using the keywords gut microbiota, spinal cord injury, and their associated subject words. The specific retrieval strategies are detailed in Supplementary Table S1.

### 2.3 Inclusion and exclusion criteria for the initial search

The same two investigators (Zaowei Zhong and Junqiao Lv) implemented study selection on an independent basis by firstly screening the titles and abstracts, followed by reviewing the full texts of eligible articles. Disagreements, if any, were resolved by consulting a third investigator (Lin Sun). Specifically, the inclusion criteria were: (1) applied an observational design (e.g., case–control study, cross-sectional study, and cohort study); (2) performed gut microbiota analysis with available data on diversity or abundance measures; and (3) included participants with spinal cord injury.

### 2.4 Potential short-chain fatty acids produced bacteria chosen

We used the retrieval method in PubMed to search for literature. We conducted a meta-analysis of identified bacteria that potential produce short-chain fatty acids by reviewing literature from establishing the database to April 7, 2024. Retrieval strategy: ((((“produced”)) AND (“bacteria”)) OR (“bacterial fermentation”)) AND (“short-chain fatty acids”)) AND (Review).

### 2.5 Eligible criteria and quality assessment

The eligible studies are as follows: (1) the samples are from patients with SCI, and 16S rRNA gene sequencing technology to elucidate the relative abundance changes of gut microbiota. (2) original research to compare the composition of gut microbiota between patients with SCI and non-SCI controls. The exclusion criteria were as follows: (1) the comparative study of microbial relative abundance between patients with SCI and non-SCI controls could not be provided; (2) There were no data on gut microbiota relative abundance. The Newcastle Ottawa scale (NOS) was used to assess the quality of case–control studies (Wells et al., 2018).

### 2.6 Outcome measures and data extraction

The primary outcomes were as follows: the relative abundance of bacteria. Two reviewers independently extracted details from the studies included in the meta-analysis. It included the first author, year of publication, location, sample size, sex, days or months from injury, injury level, injury degree, sequencing platform, and relative abundance of bacteria. However, most of the results were presented as a graph rather than an exact report of the raw data they obtained, which meant that the actual numbers had to be estimated from the data extracted from the graph. Some study results’ mean and standard deviation could not be obtained, so we used the sample’s quartile, median, maximum, minimum, and *p*-value to estimate the mean and standard deviation (Luo et al., 2018; Shi et al., 2023; Shi et al., 2020; Wan et al., 2014). Some study results included two subgroups, which we combined as one and estimated their mean and standard deviation for follow-up analyses (Altman et al., 2000). Numerical values from the graphs were estimated by GetData Graph Digitizer 2.22 software,^2^ and the study will be excluded if data were not presented or obtainable. Any controversies lead to a third reviewer settling the problem by discussion.

### 2.7 16S rRNA gene sequencing processing

Demultiplexed raw DNA sequences from the stools of SCI and non-SCI subjects from different studies were downloaded from the NCBI. The data set of subacute(PRJNA 724686) was only the SCI, and the data set of people from the same region was used as the control(PRJNA 247489, PRJNA792991). The dataset has been analyzed to obtain the mean relative abundance and SD. The raw sequencing data were imported into QIIME2/2024.01 for data processing (Bolyen et al., 2019). Due to the technical variation in the data sets included in these studies (DNA extraction kits, primers, sequencing, and platform), each data set was separately denoised and processed into amplicon sequence variants using DADA2 (Bolyen et al., 2019). The reads were trimmed that the Quartile quality score was <30. we also trimmed 21 nucleotides from the 5′ end of each read. Taxonomic classification of the operational taxonomic units (OTUs) was conducted using the classify-sklearn classification methods based on the SILVA database^3^ and the q2- feature-classifier plugin.

### 2.8 Statistical analysis

The standardized mean difference (SMD) was used as the effect indicator because the outcome indicators were all continuous variables, and point estimates and 95% confidence intervals (CI) were given for each effect measure. Statistical results were presented with a forest map. We ran *I*^2^ testing to assess the magnitude of the heterogeneity among the studies included. Suppose the heterogeneity test result was *I*^2^ ≤ 50%, meta-analysis was performed using a fixed effects model; if the heterogeneity test result was *I*^2^ > 50%, me-ta-analysis was performed using a random effects model, and the sources of heterogeneity needed to be further analyzed. After excluding obvious clinical and methodological heterogeneity, a random effects model was used for the meta-analysis (Higgins et al., 2003). Sensitivity analyses were conducted by omitting each study in turn and then rerunning the meta-analysis and assessing the differences between the results and the actual combined results.

We assessed the possibility of publication bias with a funnel plot. Egger’s tests were used to assess funnel plot asymmetry, and no significant publication bias was defined as a *p*-value >0.1. The effect of publication bias on the results of the meta-analysis was assessed using the trim-fill method (Duval and Tweedie, 2000). Egger’s tests and trim-fill method were performed with R language version 4.2.2,^4^ All graphical presentations in this study were performed with GraphPad Prism version 9,^5^ and Review Manager version 5.3 (Cochrane Collaboration).^6^

## 3 Results

### 3.1 Search flow and overview of studies

**Figure 1:**
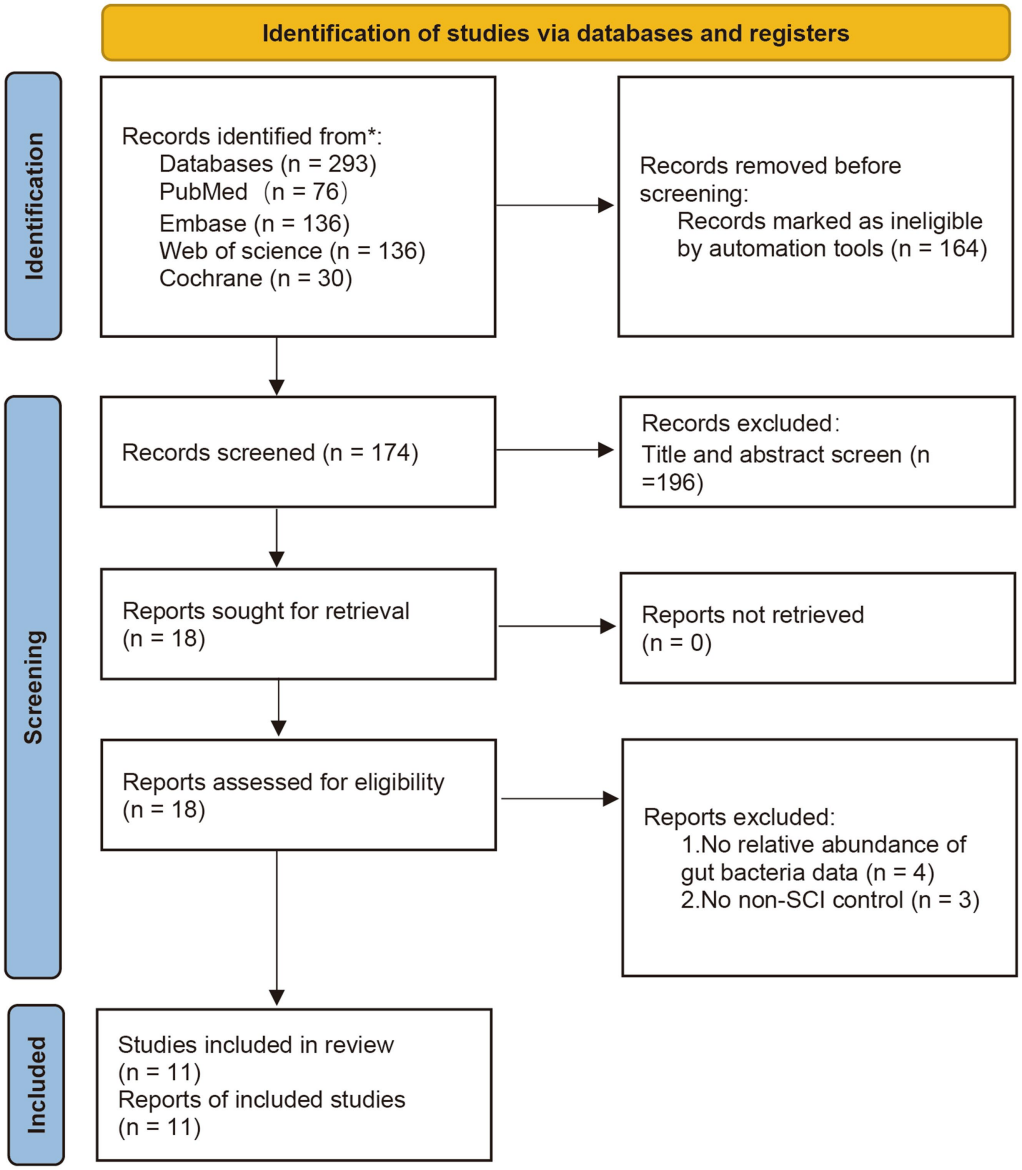
PRISMA flow diagram of study identification.

Three hundred seventy-eight studies were retrieved from PubMed, Embase, WOS, CNKI, and Cochrane Library; 164 studies marked as ineligible by automation tools were excluded, and the remaining 174 articles were evaluated. After carefully reading the titles and abstracts, 196 articles were excluded because they needed to meet the inclusion criteria. Three articles were excluded as they did not set a health control group. After reading the full texts carefully and comparing the selection criteria, 11 studies were finally included. All studies included were published between 2016 and 2024, yielding 720 individual fecal samples for microbiome analysis. Seven studies were conducted in China, one in Turkey, one in United States, one from Israel, and one in Italy. Seven studies included male and female participants, while two studies included male only (Figure 1).

All studies included in the meta-analyses were compared between patients with SCI and non-SCI controls, who were adjusted with age, and all participants without any chronic conditions. Additionally, body mass index (BMI) was matched in two studies, dietary habits were matched in four studies by all patients, and non-SCI individuals were provided standard hospital food before sample collection. Seven studies excluded the participants treated with antibiotics within 1 to 3 months before stool collection; two studies used antibiotics for inflammation in the acute stage. Five studies excluded the participants who used probiotics before enrollment. The ASIA neurological function scale of patients with SCI ranged from A to D in four studies and A, C, and D in one study: three included patients with only an A score. The rest of the studies did not specify the ASIA scores.

All studies used 16S rRNA sequencing to evaluate gut microbiota samples. Seven studies measured the V3-V4 regions, and four measured the V4 region. As for Sequencing platforms used, Illumina was used in 10 studies and microbial ecology in one study. Bacterial and archaeal rRNA databases for taxonomic assignments of sequence data in studies included are SILVA or GreenGenes. More details are shown in Table 1. The quality of case–control studies is shown in Table 2.

**Table 1:** Studies characteristics and assessments of included studies.

Study	Level of injury	SCI duration	Antibiotic use	Country	Sequencing platform, database used
Gungor et al. (2016)	Cervical, Thoracic, Lumbar	13–105 months	No	Turkey	Illumina TruSeq DNA library, GreenGenes
Zhang et al. (2018)	Cervical, Thoracic, Lumbar	6 or more months	No	China	Illumina MiSeq, SILVA, Greengenes
Zhang et al. (2019)	Cervical	6 or more months	No	China	Illumina MiSeq, SILVA, Greengenes
Lin et al. (2020)	Cervical, Thoracic, Lumbar	11 ± 2.68 months	No	China	Illumina Miseq, SILVA
Yu et al. (2021)	Thoracic	2–12 months	Yes	China	Microbial ecology platform, Greengenes
Bazzocchi et al. (2021)	Cervical, Thoracic, Lumbar	within 60 days	Yes	Italy	Illumina MiSeq, Greengenes
Pang et al. (2022)	Cervical, Thoracic, Lumbar	1–300 months	No	China	Illumina MiSeq, nucleic acid database
Li et al. (2021)	Cervical, Thoracic, Lumbar	4 days-53 years	Yes	America	Illumina Miseq, SILVA
Kong et al. (2023)	Cervical, Thoracic	22.81 ± 1.15 months	Yes	China	Illumina MiSeq/HiSeq
Jing et al. (2023)	Cervical, Thoracic, Lumbar	2-70 month	no	China	Illumina Miseq, SILVA
Gur Arie et al. (2024)	Cervical, Thoracic, Lumbar	60-259 days	Yes	Israel	Illumina MiSeq, Greengenes

**Table 2:** The Newcastle Ottawa scale scores of studies included.

Studies	Adequate definition of cases	Representativeness of the cases	Selection of controls	Definition of controls	Comparability control for important factor	Ascertainment of exposure	Same method of ascertainment for cases and controls	Nonresponse rate	Scores
Zhang et al. (2018)	★	★	☆	★	★★	★	★	★	8
Li et al. (2022)	★	★	★	★	★	★	★	★	8
Lin et al. (2020)	★	★	★	★	★	★	★	★	8
Gungor et al. (2016)	★	★	☆	★	★	★	★	★	7
Pang et al. (2022)	★	★	☆	★	★	★	★	★	7
Kong et al. (2023)	★	★	☆	★	★	★	★	★	7
Yu et al. (2021)	★	★	★	★	★★	★	★	★	9
Zhang et al. (2019)	★	★	☆	★	★	★	★	★	7
Bazzocchi et al. (2021)	★	★	☆	★	★	★	★	★	7
Gur Arie et al. (2024)	★	★	☆	★	★	★	★	★	7
Jing et al. (2023)	★	★	☆	★	★	★	★	★	7

### 3.2 Bacteria selected for meta-analyses

We searched for literature and summarized the bacterial genera that potential produce SCFAs (Dalile et al., 2019; Dicks, 2022; Vacca et al., 2020). Summarized in Table 3.

**Table 3:** Bacteria selected for meta-analysis.

Bacteria	Reason to meta
Fimicutes	Produce high amounts of butyrate; butyrate can reduce neuroinflammatory responses.
Bacteroidetes	Produce high levels of acetate and propionate; both have anti-inflammatory properties.
Clostridiales	Correlates with locomotor recovery in SCI mice.
Atopobium	The relative abundance of Atopobium in the gut increases in patients with colitis who take rifaximin, and rifaximin has been observed to have neuroprotective effects in mice with craniocerebral injury.
Bacteroides	Produces neurotransmitters such as GABA. Converts tryptophan in food to 5-HT.
Bifidobacterium	Correlates with butyric acid and valeric acid in SCI patients and converts tryptophan in food to 5-HT.
Blautia	It can convert polymethoxyflavones with various biological functions, such as anti-inflammatory and neuroprotective activities.
Coprococcus	Positively correlated with tryptophan metabolites, affecting the host’s intestinal barrier function and antioxidant activity.
Dorea	A positive correlation between protein 1 receptor and cytotoxic T lymphocyte-associated protein 4 blockade, along with increased levels of Dorea, has been reported.
Eubacterium	Butyrate is the main SCFA produced by the Roseburia/*Eubacterium rectale* group, especially at a mildly acidic pH, along with the consumption of acetate.
Faecalibacterium	Correlates with acetic acid, propionic acid, isobutyric acid, butyric acid, and isovaleric acid in SCI patients.
Lachnospira	Pectin-utilizing Firmicutes species.
Lactobacillus	Produces neurotransmitters such as GABA and converts tryptophan in food to 5-HT.
Prevotella	Converts tryptophan in food to 5-HT.
Roseburia	Butyrate is the main SCFA produced by the Roseburia/*Eubacterium rectale* group, especially at a mildly acidic pH, along with the consumption of acetate.Converts tryptophan in food to 5-HT.
Ruminococcus	Correlates with acetic acid, butyric acid, and valeric acid in SCI patients.

2 phyla, 1 order, and 14 genus.

### 3.3 Gut bacteria changed of SCI subacute and chronic stage

We analyzed the PRJNA724686 dataset, a sample collected from 21 to 36 days after SCI. We identify the data as subacute. The injury time and sampling time of the study population included in the meta-analyses range from 2 months to 10 years, and we consider it the chronic phase.

**Figure 2:**
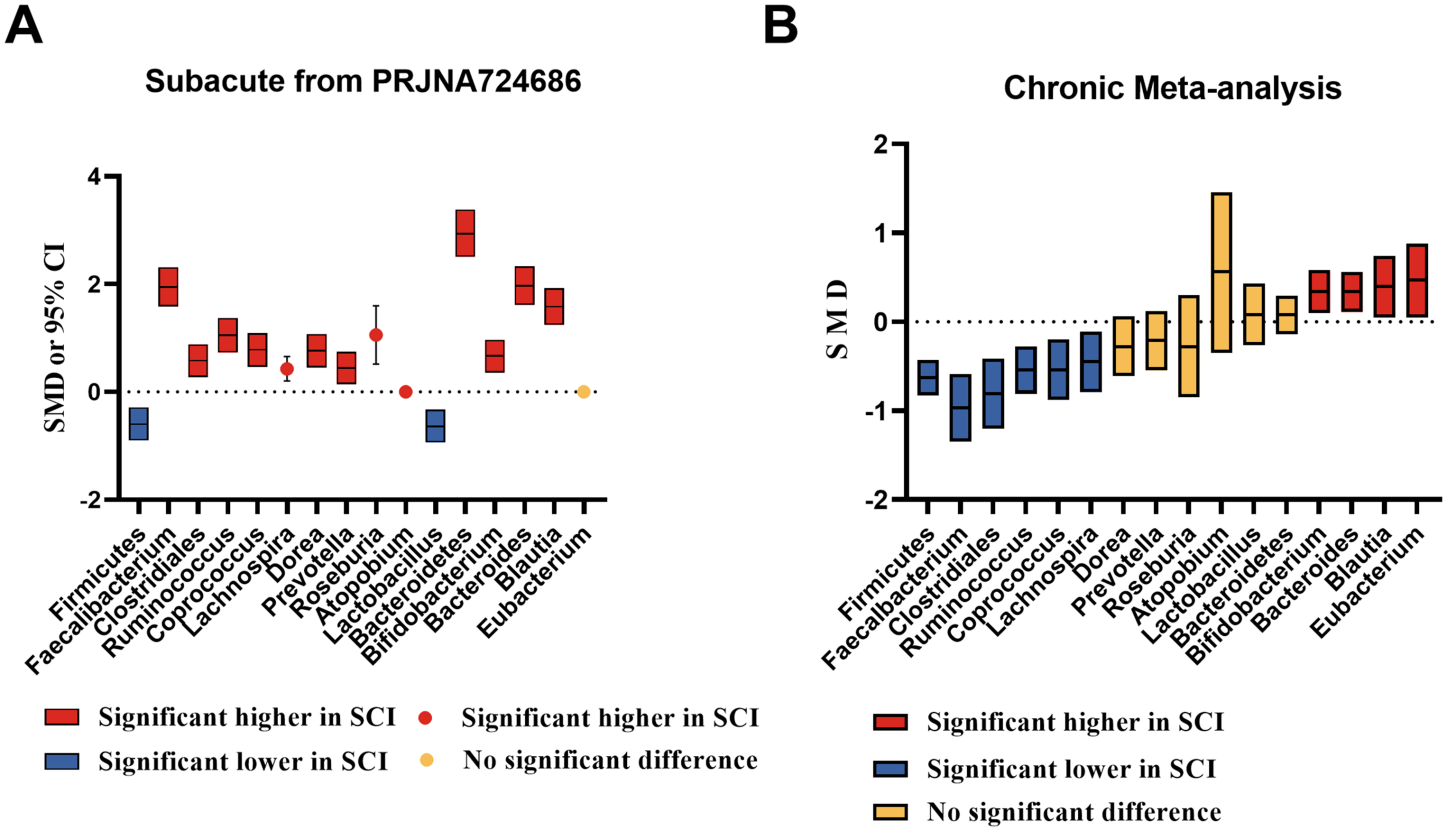
SCFAs-producing bacteria changed of SCI subacute and chronic stage **(A)** Subacute phase. **(B)** Chronic phase. SMD, standard mean differences; CI, confidence interval.

In patients with SCI during the subacute phase, the relative abundance of Firmicutes and Lactobacillus decreases. In contrast, the relative abundance of Bacteroidetes, Bacteroides, Blautia, Clostridales, Faecalbacterium, Ruminococcus, Coprococcus, Lachnospira, Dorea, Prevotella, Roseburia, Atopobium, and Bifidobacterium increases. There is no significant difference in Eubacteria (Figure 2).

**Figure 3:**
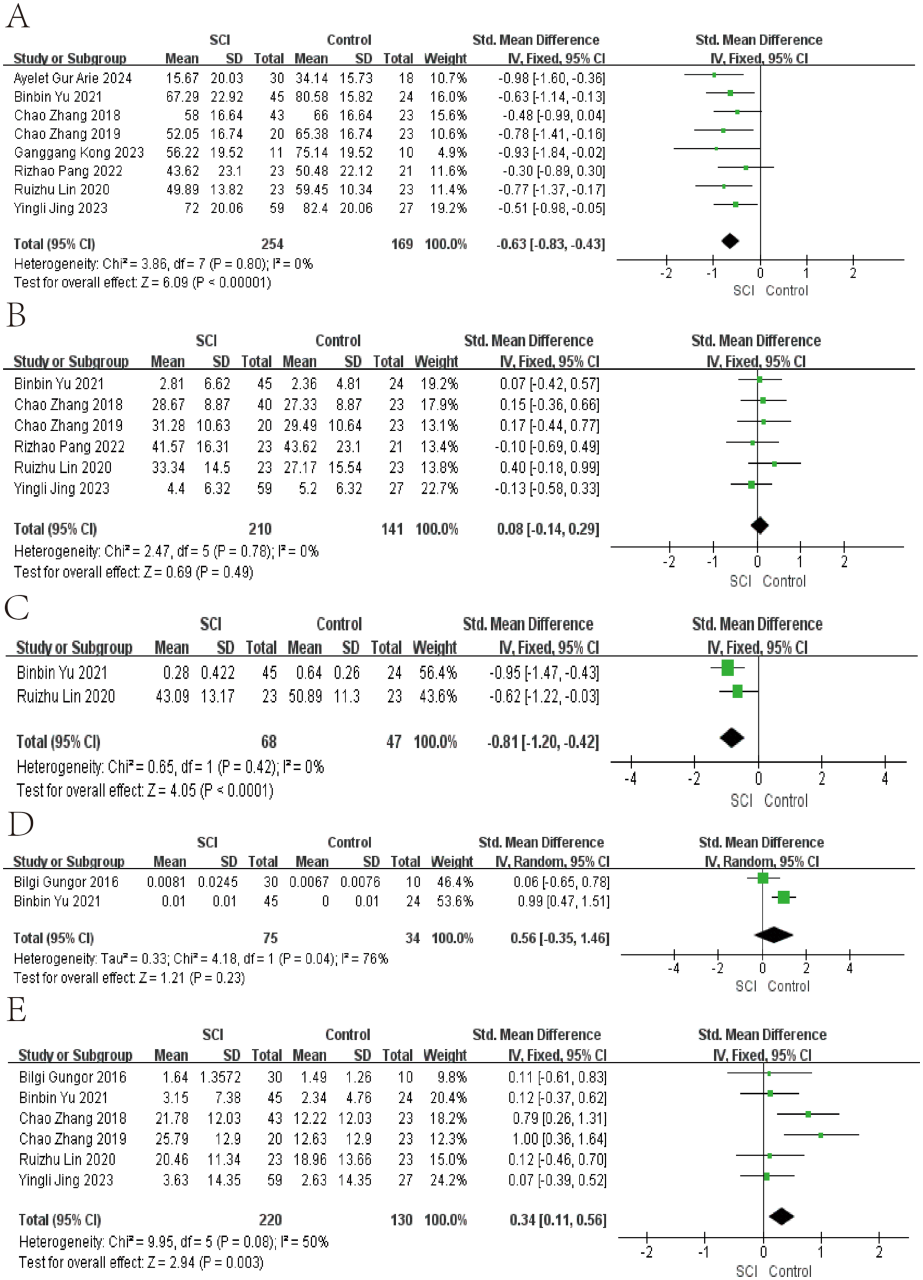
Forest map of Relative abundance of bacteria in gut between SCI and non-SCI control. **(A)** Firmicutes, **(B)** Bacteroidetes, **(C)** Clostridiales, **(D)** Atopobium, and **(E)** Bacteroides. SMD, standard mean differences; CI, confidence interval.

**Figure 4:**
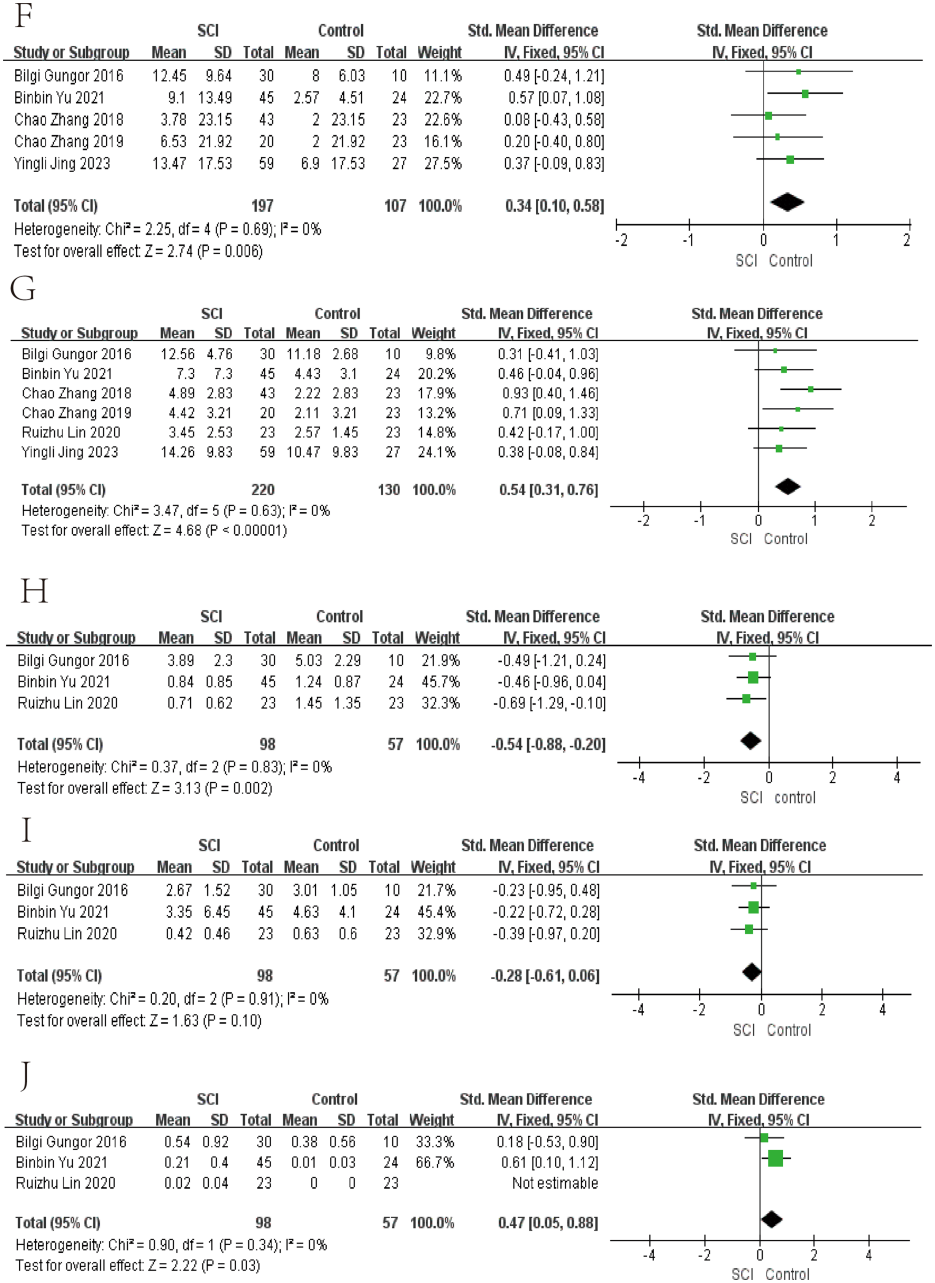
Forest map of Relative abundance of bacteria in gut between SCI and non-SCI control. **(F)** Bifidobacterium, **(G)** Blautia, **(H)** Coprococcus, **(I)** Dorea, and **(J)** Eubacterium. SMD, standard mean differences; CI, confidence interval.

**Figure 5:**
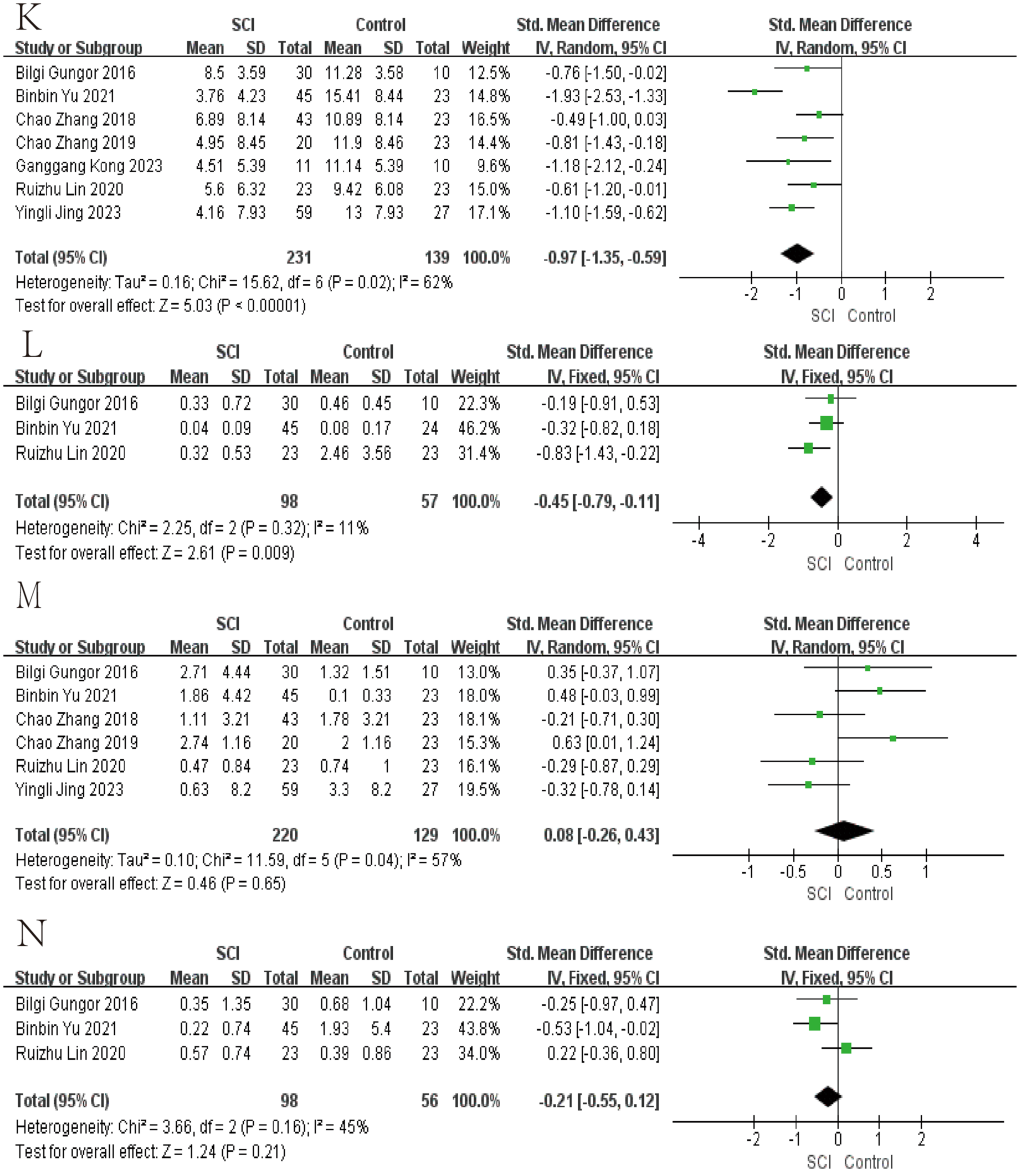
Forest map of Relative abundance of bacteria in gut between SCI and non-SCI control. **(K)** Faecalibacterium, **(L)** Lachnospira, **(M)** Lactobacillus, and **(N)** Prevotella. SMD, standard mean differences; CI, confidence interval.

**Figure 6:**
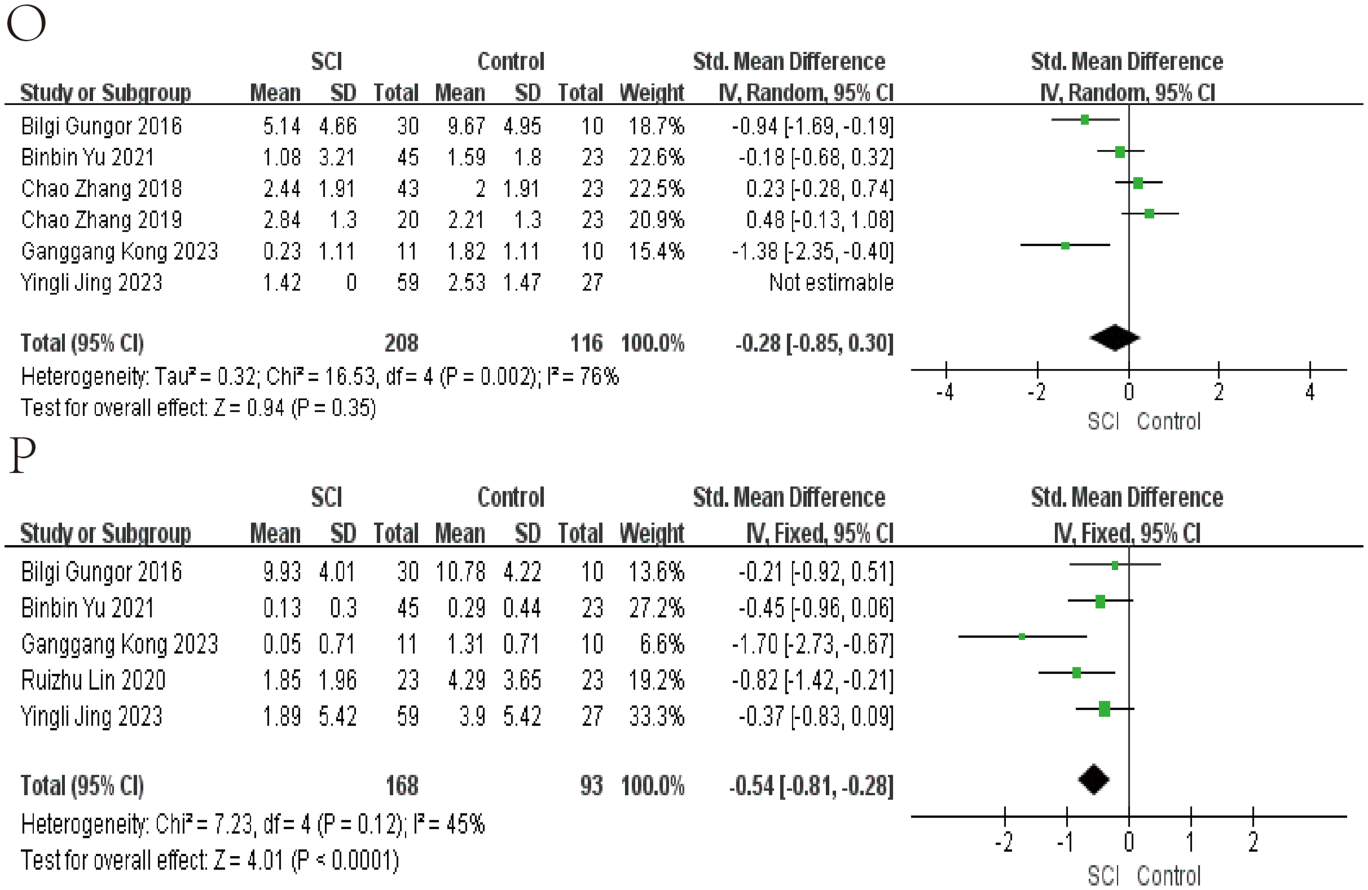
Forest map of Relative abundance of bacteria in gut between SCI and non-SCI control. **(O)** Roseburia and **(P)** Ruminococcus. SMD, standard mean differences; CI, confidence interval.

In the meta-analyses of patients with chronic SCI, the number of Firmicutes decreased in the SCI group, while there was no significant difference in the number of Bacteroidetes. The average abundance proportion of Bifidobacterium, Bacteroides, Blautia, and Eubacterium in SCI patients is higher, with statistical differences. In contrast, the abundance of Clostridiales, Ruminococcus, Feacalbacterium, Coprococcus, and Lachnospira in SCI patients is lower, achieving statistical differences. There was no significant difference in Dorea, Prevotella, Roseburia, Atopobium, and *Lactobacillus* (Figures 3–6).

### 3.4 Publication bias and heterogeneity

**Figure 7:**
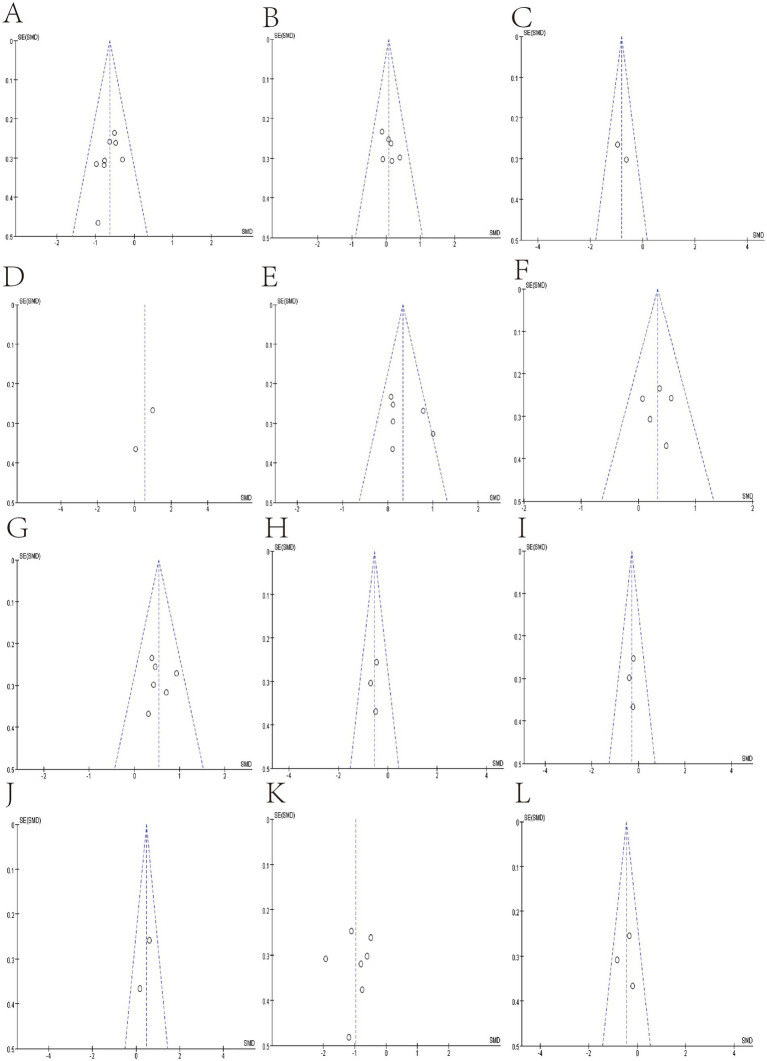
Funnel plot of Relative abundance of bacteria in gut between SCI and non-SCI control. **(A)** Firmicutes, **(B)** Bacteroidetes, **(C)** Clostridiales, **(D)** Atopobium, **(E)** Bacteroides. **(F)** Bifidobacterium, **(G)** Blautia, **(H)** Coprococcus, **(I)** Dorea, **(J)** Eubacterium, **(K)** Faecalibacterium, and **(L)** Lachnospira,

**Figure 8:**
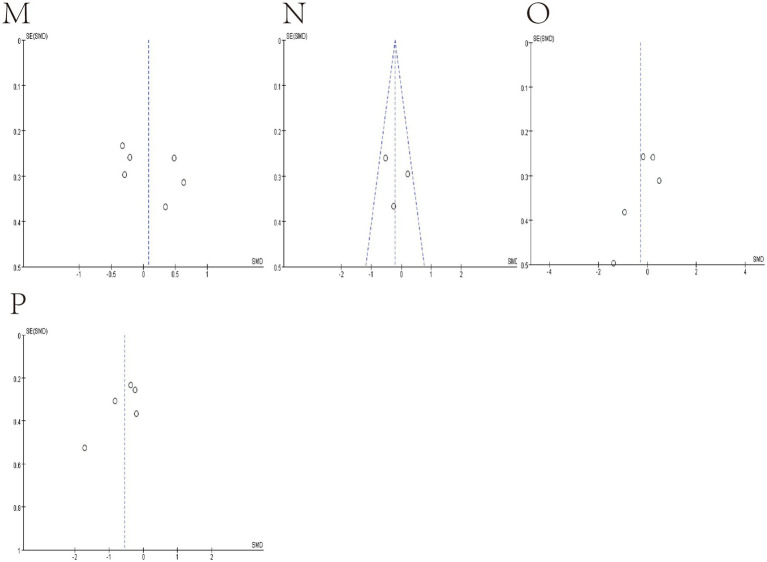
Funnel plot of Relative abundance of bacteria in gut between SCI and non-SCI control. (M) Lactobacillus, (N) Prevotella, (O) Roseburia and (P) Ruminococcus.

We use funnel plots and Egger’s test to evaluate publication bias (Figures 7, 8; Table 4). We observed a phenomenon in the meta-analyses of the genera Bacteroidetes, Bluatia, and Bifidobacterium, where there was one study in their funnel plots, and the effectiveness of the study was outside the dashed range of the funnel plots. We consider that the results of these studies beyond the dashed range may have significant biases or anomalies, which may be due to methodological issues, data quality considerations, or other potential factors (Supplementary Figures S1, S2). We paid special attention to this study and tested its impact on the overall results in subsequent sensitivity analysis. Considering their significant bias in the meta-analyses of bacterial abundance, we have decided to exclude them. Then, a meta-analysis was conducted again, and it was found that heterogeneity was significantly reduced (Figures 3, 4) After Egger’s test, there are public biases in Bacteroides and Prevotella. The trim-fill shows that bias does not affect the meta-analyses result. The rest of the bacteria were not found in public bias. After applying the trim-fill method, the meta of Bacteroides changed direction and showed no significant difference. The meta-analysis of Prevotella did not show significant differences after using the trim-fill method, and there is no evidence of publication bias (Table 4; Supplementary Figure S3).

**Table 4:** Egg’s test results of bacteria.

Bacteria	*p* value of Egg’s test
Firmicutes	0.7672
Bacteroidetes	0.3081
Atopobium	–
Bacteriodes	0.0943
Bifidobacterium	0.3694
Blautia	0.6368
Clostridiales	–
Coprococcus	0.3488
Dorea	0.181
Faecalbacterium	0.3241
Lachnospira	0.3425
Lactobacillus	0.8412
Prevotella	0.0959
Roseburia	0.235
Ruminococcus	0.11

We performed sensitivity analyses by omitting each study, and the results were stable, except Bacteroides and Eubacterium were unstable. At the same time, we also summarized the studies that significantly impacted heterogeneity in the meta-analyses of the relative abundance of various bacteria (Table 5). The study by Zhang et al. (2018) significantly impacts the *p*-value results of Bacteroides, which may be due to the higher relative abundance of genus Bacteroides in the population with ASIA grade A SCI in the chronic phase compared to other grades. ASIA grade may be a source of heterogeneity (Zhang et al., 2018).

**Table 5:** Studies that significantly impacted heterogeneity or *p*- value in the meta-analysis.

Bacteria	Heterogeneity (over 30%)	*p*- value (changed over 0.05)
Bacteroides	–	Zhang et al. (2018)
Bifidobacterium	Lin et al. (2020)	Lin et al. (2020)
Blautia	Kong et al. (2023)	Kong et al. (2023)
Eubacterium	–	Yu et al. (2021)
Faecalbacterium	Yu et al. (2021)	–
Ruminococcus	Kong et al. (2023)	–

The meta-analysis of Eubacterium showed no significant difference after excluding studies by Yu et al. (2021) which may be related to their study only including the causes of thoracic spinal cord injury. In Blautia meta-analysis, the study by Kong et al. showed the opposite direction compared to others (Kong et al., 2023). After exclusion, it was found that there was a significant difference in the meta-analysis results and a significant reduction in heterogeneity. After comparing it with other studies, it was found that the study of Kong et al. population used antibiotics. Antibiotics may be a source of heterogeneity and bias, and antibiotics may significantly influence the relative abundance of Blautia. After conducting a sensitivity analysis in Ruminococcus, significant heterogeneity was observed in the study by Kong et al., but it did not alter the results of the meta-analysis. After incorporating study of Lin et al. into the meta-analysis of Bifidobacterium, it was found that there was a significant increase in heterogeneity and a change in the results of the meta-analysis, with a significant increase in p-value. We found bias in the funnel plot and compared it with the results of other studies. We found that research of Lin et al. direction was the opposite, and the different lengths of injury time may be the reason (Lin et al., 2020). Research of Yu et al. contributed to all heterogeneity in the meta-analysis of Faecalbacterium, and we speculate that the source of heterogeneity is the damaged segment. For the meta-analysis of Prevotella, studies from Yu et al. and Lin et al. significantly influenced heterogeneity, and the use of antibiotics or damaged segments may affect the relative abundance of Prevotella. Clostridiales did not observe significant heterogeneity (Table 4; Figures 3–6).

## 4 Discussion

This study assessed gut microbiota alterations across a spectrum of SCI through meta-analyses. The main findings were: (1) In subacute phase of spinal cord injury, the relative abundance of Bacteroidetes, Clostridiales, Faecalbacterium, Ruminococcus, Coprococcus, Lachnospira, Dorea, Prevotella, Roseburia, Atopobium, Bifidobacterium, Bacteroides, and Blautia increased. Firmicutes and Lactobacillus decreased. (2) In chronic phase. Firmicutes decreased in the SCI group, and Bacteroidetes showed no significant difference. Bifidobacterium, Bacteroides, Blautia, and Eubacterium were found to have a higher average proportion of abundance in patients with SCI compared to non-SCI persons, and Clostridiales, Ruminococcus, Faecalbacterium, Coprococcus, and Lachnospira showed a lower abundance in SCI; statistical differences were reached. Dorea, Prevotella, Roseburia, Atopobium, and Lactobacillus found no significant difference.

Substantial heterogeneity was not observed in this study. The composition of gut microbiota among individuals was influenced by age, gender, diet, genes, and environment. We evaluated all included studies and found that some of these studies differ in several ways, including sex, SCI duration, level of injury, ASIA scores, country design, and database used, which contribute to heterogeneity in meta-analyses of some bacteria. But overall, heterogeneity is acceptable. Egger’s test indicated the presence of publication bias. The trim-and-fill method was used to assess the impact of this bias on Bacteroides and Prevotella. The results showed that the bias had no significant effect on Bacteroides, but it did affect Prevotella. Therefore, no meta-analysis was conducted on Prevotella. The source of publication bias for Prevotella may be related to population-specific gut microbiota profiles, where studies may have been unpublished due to the lack of significant differences observed after spinal cord injury (Arumugam et al., 2011).

Multiple studies on SCI in mice have found a negative correlation between Clostridiales and BMS, so we have paid extra attention to this (He et al., 2022; Jing et al., 2019; Kigerl et al., 2016). Zhang et al. (2023) examined the diversity and relative abundance of gut microbiota in mice with SCI. They found that the Clostridiales significantly increased in the mice gut after SCI. However, currently published studies on the gut microbiota of SCI mice are acute or subacute-phase samples, unlike human samples, which are mainly chronic-phase samples (Gungor et al., 2016; Jing et al., 2023; Yu et al., 2021; Zhang et al., 2018). In this study, the Clostridiales increased in the subacute phase and decreased in the chronic phase. Further research is needed to determine whether the increase in Clostridiales during the acute phase and decrease during the chronic phase after SCI affects the recovery of motor function in mice or humans after SCI. In the meta-analysis by Zhang et al., there was no significant difference in the relative abundance of Firmicutes, Bacteroidetes, Bacteroides, and Lactobacillus compared to the sham surgery group, which may be due to the high heterogeneity (greater than 70) in the included studies. Because in multiple studies on spinal cord injury in humans and mice, it has been observed that the content of SCFAs in feces is lower than that in the control group, the SCFAs-producing bacteria in the gut was likely reduced (Jing et al., 2023; Kigerl et al., 2016).

Firmicutes and Bacteroidetes are dominant bacteria in the human gut, and their relative abundance rapidly changes, leading to gut dysbiosis (Arumugam et al., 2011). In subacute phase of spinal cord injury, data showed a decrease in the relative abundance of Firmicutes compared to the non-spinal cord injury control group, an increase in the relative abundance of Bacteroidetes. In chronic phase, the relative abundance of Firmicutes decreased, and there was no significant difference in the relative abundance of Bacteroidetes compared to the control group without spinal cord injury. Gut dysbiosis has been observed in the population’s subacute and chronic phases.

For the overall gut microbiota, the genus of potential SCFAs-producing bacteria is lower in the chronic phase of spinal cord injury than in the subacute phase, and gut dysbiosis is present in both the subacute and chronic phases. The gut microbiota has coexisted with the host for a long time; recently, some have regarded the gut microbiota as an endocrine organ of the host (Jogia and Ruitenberg, 2020; Turroni et al., 2018). We speculate that the general increase in the relative abundance of SCFAs-producing bacterial genera during the subacute phase of spinal cord injury is a compensatory response of the gut microbiota to the injury, while in the chronic phase, there is a certain degree of decompensation. The decrease in regenerative potential from the intestine may result in partial loss of motor function recovery. For Lactobacillus, a study using melatonin to treat mice with spinal cord injury found that after administration, the relative abundance of Lactobacillus was positively correlated with BMS and negatively correlated with FITC-dextran permeability, and an increase in the molecules Occludin and ZO-1 associated with intestinal permeability was also observed (Jing et al., 2019). An earlier animal study found that after spinal cord injury, the intestinal permeability of mice increased, and bacteria in the gut entered and spread with the bloodstream, activating pathological immune responses in the gut-associated lymphoid tissues, while these bacteria translocated throughout the body caused systemic inflammation. Supplementation of probiotics rich in Lactobacillus in mice with spinal cord injury was observed to reduce pathological immune responses in intestinal mucosal lymph nodes, mainly through activation of regulatory T lymphocytes. Lactobacillus may benefit mice with spinal cord injury by reducing the translocation of intestinal bacteria and regulating the immune response in the intestinal mucosal lymph nodes (Kigerl et al., 2016). During the subacute phase, the abundance of Lactobacillus decreases, possibly because it is more prone to leakage from the intestine, thereby affecting the recovery of host motor function. The impact of Lactobacillus on spinal cord injury deserves further research.

The role of SCFAs in the nervous system is increasingly being revealed through research. After supplementation with SCFA in mice with spinal cord injury, it was observed that astrocyte proliferation decreased, microglial activation was inhibited, NF- *κ* B signal transduction was downregulated, and lower levels of neuroinflammation and better motor recovery compared to the sham surgery group (Jing et al., 2023). In a mouse model of chronic cerebral hypoperfusion, SCFAs inhibit the NF-κB pathway and activate the Erk1/2 cascade, subsequently reducing neuroinflammation and neuronal apoptosis in the hippocampus after injury (Xiao et al., 2022). After atorvastatin treatment, Firmicutes and Lactobacillus increased, Bacteroidetes decreased, and neuroinflammation of ischemic stroke mice was attenuated (Zhang et al., 2021). 10-strain isolated from the infant’s gut as a probiotic cocktail to treat mice modulated the gut microbiome, increased SCFA production, and ameliorated gut microbiome dysbiosis (Nagpal et al., 2018). SCFAs can reach the whole body through the blood, cross the blood–brain barrier, and exert neuroprotective effects, which is an important part of understanding the brain-gut axis (Mitchell et al., 2011).

Our study verified the overall change trend of SCFAs-producing bacteria in human samples during chronic spinal cord injury through meta-analysis. SCFAs-producing bacteria play an essential role in the brain-gut axis and have been shown in multiple animal studies to promote neuromotor function recovery, so increasing the abundance of these bacteria in the gut or avoiding their decline as much as possible (more cautious use of antibiotics) may benefit motor function recovery after spinal cord injury. We hope that our results will provide reference and theoretical support for targeting gut microbiota in patients with spinal cord injury.

## 5 Conclusion

The genus of potential SCFAs-producing bacteria is lower in the chronic phase of spinal cord injury than in the subacute phase, and gut dysbiosis is present in both the subacute and chronic phases.

## Data Availability

The original contributions presented in the study are included in the article/Supplementary material, further inquiries can be directed to the corresponding author/s.

## Footnotes

^1^ https://www.crd.york.ac.uk/prospero/

^2^ http://www.getdata-graph-digitizer.com/

^3^ https://data.qiime2.org/2024.01/common/silva-138-99-nb-classifier.qza

^4^meta packages https://www.r-project.org/

^5^ https://www.graphpad-prism.cn

^6^ https://training.cochrane.org/online-learning/core-software/revman

## Supplementary material

Supplementary Figure S1 Funnel plot of no studies excluded. **(A)** Bacteroidetes, **(B)** Blautia, and **(C)** Bifidobacterium.

Supplementary Figure S2 Forest map of no studies excluded. **(A)** Bacteroidetes, **(B)** Blautia, and **(C)** Bifidobacterium.

Supplementary Figure S3 Trim-fill method result of **(A)** Bacteroides and **(B)** Prevotella.

Supplementary Table S1 Retrieval strategies of this study.
