# Supplementary material for: Changes of potential shorty-chain fatty acids producing bacteria in the gut of patients with spinal cord injury: a systematic review and meta-analysis
Source: Front Microbiol. 2025 Feb 27;16:1483794. doi: 10.3389/fmicb.2025.1483794 (PMC11905530; doi:10.3389/fmicb.2025.1483794)
Supplement: Supplementary Figure S1 — Funnel plot of no studies excluded. (A) Bacteroidetes, (B) Blautia, and (C) Bifidobacterium. [file Supplementary_file_1.zip › data sheet 1/Supplemental table 1.DOCX]

| **PubMed** searched on March 31, 2024 | Results |
| --- | --- |
| #1Search: (Spinal Cord Injuries[MeSH]) OR ((((((((((((((((((((((((((((((((((((((Spinal Cord Trauma[Title/Abstract]) OR (Cord Trauma, Spinal[Title/Abstract])) OR (Cord Traumas, Spinal[Title/Abstract])) OR (Spinal Cord Traumas[Title/Abstract])) OR (Trauma, Spinal Cord[Title/Abstract])) OR (Traumas, Spinal Cord[Title/Abstract])) OR (Myelopathy, Traumatic[Title/Abstract])) OR (Myelopathies, Traumatic[Title/Abstract])) OR (Traumatic Myelopathies[Title/Abstract])) OR (Traumatic Myelopathy[Title/Abstract])) OR (Injuries, Spinal Cord[Title/Abstract])) OR (Cord Injuries, Spinal[Title/Abstract])) OR (Cord Injury, Spinal[Title/Abstract])) OR (Injury, Spinal Cord[Title/Abstract])) OR (Spinal Cord Injury[Title/Abstract])) OR (Spinal Cord Transection[Title/Abstract])) OR (Cord Transection, Spinal[Title/Abstract])) OR (Cord Transections, Spinal[Title/Abstract])) OR (Spinal Cord Transections[Title/Abstract])) OR (Transection, Spinal Cord[Title/Abstract])) OR (Transections, Spinal Cord[Title/Abstract])) OR (Spinal Cord Laceration[Title/Abstract])) OR (Cord Laceration, Spinal[Title/Abstract])) OR (Cord Lacerations, Spinal[Title/Abstract])) OR (Laceration, Spinal Cord[Title/Abstract])) OR (Lacerations, Spinal Cord[Title/Abstract])) OR (Spinal Cord Lacerations[Title/Abstract])) OR (Post-Traumatic Myelopathy[Title/Abstract])) OR (Myelopathies, Post-Traumatic[Title/Abstract])) OR (Myelopathy, Post-Traumatic[Title/Abstract])) OR (Post Traumatic Myelopathy[Title/Abstract])) OR (Post-Traumatic Myelopathies[Title/Abstract])) OR (Spinal Cord Contusion[Title/Abstract])) OR (Contusion, Spinal Cord[Title/Abstract])) OR (Contusions, Spinal Cord[Title/Abstract])) OR (Cord Contusion, Spinal[Title/Abstract])) OR (Cord Contusions, Spinal[Title/Abstract])) OR (Spinal Cord Contusions[Title/Abstract])) Sort by: Publication Date  #2Search: ("Gastrointestinal Microbiome"[Mesh]) OR (((((((((((((((((((((((((((((((((((((Gastrointestinal Microbiomes[Title/Abstract]) OR (Microbiome, Gastrointestinal[Title/Abstract])) OR (Gut Microbiome[Title/Abstract])) OR (Gut Microbiomes[Title/Abstract])) OR (Microbiome, Gut[Title/Abstract])) OR (Gut Microflora[Title/Abstract])) OR (Microflora, Gut[Title/Abstract])) OR (Gut Microbiota[Title/Abstract])) OR (Gut Microbiotas[Title/Abstract])) OR (Microbiota, Gut[Title/Abstract])) OR (Gastrointestinal Flora[Title/Abstract])) OR (Flora, Gastrointestinal[Title/Abstract])) OR (Gut Flora[Title/Abstract])) OR (Flora, Gut[Title/Abstract])) OR (Gastrointestinal Microbiota[Title/Abstract])) OR (Gastrointestinal Microbiotas[Title/Abstract])) OR (Microbiota, Gastrointestinal[Title/Abstract])) OR (Gastrointestinal Microbial Community[Title/Abstract])) OR (Gastrointestinal Microbial Communities[Title/Abstract])) OR (Microbial Community, Gastrointestinal[Title/Abstract])) OR (Gastrointestinal Microflora[Title/Abstract])) OR (Microflora, Gastrointestinal[Title/Abstract])) OR (Gastric Microbiome[Title/Abstract])) OR (Gastric Microbiomes[Title/Abstract])) OR (Microbiome, Gastric[Title/Abstract])) OR (Intestinal Microbiome[Title/Abstract])) OR (Intestinal Microbiomes[Title/Abstract])) OR (Microbiome, Intestinal[Title/Abstract])) OR (Intestinal Microbiota[Title/Abstract])) OR (Intestinal Microbiotas[Title/Abstract])) OR (Microbiota, Intestinal[Title/Abstract])) OR (Intestinal Microflora[Title/Abstract])) OR (Microflora, Intestinal[Title/Abstract])) OR (Intestinal Flora[Title/Abstract])) OR (Flora, Intestinal[Title/Abstract])) OR (Enteric Bacteria[Title/Abstract])) OR (Bacteria, Enteric[Title/Abstract])) Sort by: Publication Date  #3 #1 AND #2 | 80624  95865  76 |
| **Embase** searched on March 31, 2024 |  |
| #1'injury, spinal cord':ab,ti OR 'spinal cord injuries':ab,ti OR 'spinal cord trauma':ab,ti OR 'trauma, spinal cord':ab,ti OR 'spinal cord injury'  #2'alimentary canal flora':ab,ti OR 'alimentary tract flora':ab,ti OR 'bowel flora':ab,ti OR 'bowel microbiota':ab,ti OR 'digestive canal flora':ab,ti OR 'digestive tract flora':ab,ti OR 'enteric flora':ab,ti OR 'enteric microbiota':ab,ti OR 'flora, intestine':ab,ti OR 'gastro intestinal flora':ab,ti OR 'gastrointestinal canal flora':ab,ti OR 'gastrointestinal flora':ab,ti OR 'gastrointestinal microbiome':ab,ti OR 'gastrointestinal microbiota':ab,ti OR 'gastrointestinal tract flora':ab,ti OR 'gut bacteria':ab,ti OR 'gut microbiota':ab,ti OR 'intestinal bacteria':ab,ti OR 'intestinal canal flora':ab,ti OR 'intestinal flora':ab,ti OR 'intestinal microbiota':ab,ti OR 'intestinal microflora':ab,ti OR 'intestinal microorganism':ab,ti OR 'intestinal tract flora':ab,ti OR 'intestine bacteria':ab,ti OR 'intestine bacteria change':ab,ti OR 'intestine bacterial flora':ab,ti OR 'intestine bacterium':ab,ti OR 'intestine microbial flora':ab,ti OR 'intestine microflora':ab,ti OR ‘gastrointestine flora':ab,ti OR ‘gastrointestine tract flora':ab,ti OR 'intestinal bacterial flora':ab,ti OR 'intestinal bacterium':ab,ti OR 'intestinal microbe':ab,ti OR 'intestinal microbes':ab,ti OR 'intestine flora'  #3 #1 AND #2 | 88170  14892  136 |
| **Web of science** searched on March 31, 2024 |  |
| #1 TS=(Spinal cord injury OR Spinal injury OR Spinal Cord Trauma OR Spinal Cord Transection OR Spinal Cord Laceration OR Post-Traumatic Myelopathy OR Spinal Cord Contusion OR Spinal Cord Injuries)  #2 TS=( Gastrointestinal Microbiomes OR Microbiome, Gastrointestinal OR Gut Microbiome OR Gut Microbiomes OR Microbiome, Gu OR Gut Microflora OR Microflora, Gut OR Gut Microbiota OR Gut Microbiotas OR Microbiota, Gut OR Gastrointestinal Flora OR Flora, Gastrointestinal OR Gut Flora OR Flora, Gut OR Gastrointestinal Microbiota OR Gastrointestinal Microbiotas OR Microbiota, Gastrointestinal OR Gastrointestinal Microbial Community OR Gastrointestinal Microbial Communities OR Microbial Community, Gastrointestinal OR Gastrointestinal Microflora OR Microflora, Gastrointestinal OR Gastric Microbiome OR Gastric Microbiomes OR Microbiome, Gastric OR Intestinal Microbiome OR Intestinal Microbiomes OR Microbiome, Intestinal OR Intestinal Microbiota OR Intestinal Microbiotas OR Microbiota, Intestinal OR Intestinal Microflora OR Microflora, Intestinal OR Intestinal Flora OR Flora, Intestinal OR Enteric Bacteria OR Bacteria, Enteric OR Gastrointestinal Microbiome) | 65982  122454 |
| #3 #1 AND #2 | 136 |
| **Cochrane** searched on March 31, 2024 |  |
| #1 MeSH descriptor: [Spinal Cord Injuries] explode all trees  Spinal Cord Trauma OR Cord Trauma, Spinal OR Cord Traumas, Spinal OR Spinal Cord Traumas OR Trauma, Spinal Cord OR Traumas, Spinal Cord OR Myelopathy, Traumatic OR Myelopathies, Traumatic OR Traumatic Myelopathies OR Traumatic Myelopathy OR Injuries, Spinal Cord OR Cord Injuries, Spinal OR Cord Injury, Spinal OR Injury, Spinal Cord OR Spinal Cord Injury OR Spinal Cord Transection OR Cord Transection, Spinal OR Cord Transections, Spinal OR Spinal Cord Transections OR Transection, Spinal Cord OR Transections, Spinal Cord OR Spinal Cord Laceration OR Cord Laceration, Spinal OR Cord Lacerations, Spinal OR Laceration, Spinal Cord OR Lacerations, Spinal Cord OR Spinal Cord Lacerations OR Post-Traumatic Myelopathy OR Myelopathies, Post-Traumatic OR Myelopathy, Post-Traumatic OR Post Traumatic Myelopathy OR Post-Traumatic Myelopathies OR Spinal Cord Contusion OR Contusion, Spinal Cord OR Contusions, Spinal Cord OR Cord Contusion, Spinal OR Cord Contusions, Spinal OR Spinal Cord Contusions  #2 MeSH descriptor: [Gastrointestinal Microbiome] explode all trees  Gastrointestinal Microbiomes OR Microbiome, Gastrointestinal OR Gut Microbiome OR Gut Microbiomes OR Microbiome, Gu OR Gut Microflora OR Microflora, Gut OR Gut Microbiota OR Gut Microbiotas OR Microbiota, Gut OR Gastrointestinal Flora OR Flora, Gastrointestinal OR Gut Flora OR Flora, Gut OR Gastrointestinal Microbiota OR Gastrointestinal Microbiotas OR Microbiota, Gastrointestinal OR Gastrointestinal Microbial Community OR Gastrointestinal Microbial Communities OR Microbial Community, Gastrointestinal OR Gastrointestinal Microflora OR Microflora, Gastrointestinal OR Gastric Microbiome OR Gastric Microbiomes OR Microbiome, Gastric OR Intestinal Microbiome OR Intestinal Microbiomes OR Microbiome, Intestinal OR Intestinal Microbiota OR Intestinal Microbiotas OR Microbiota, Intestinal OR Intestinal Microflora OR Microflora, Intestinal OR Intestinal Flora OR Flora, Intestinal OR Enteric Bacteria OR Bacteria, Enteric OR Gastrointestinal Microbiome  #3 #1 AND #2 in Trials | 13056  9534  30 |
