# Supplementary figures and images for: Changes of potential shorty-chain fatty acids producing bacteria in the gut of patients with spinal cord injury: a systematic review and meta-analysis
Source: Front Microbiol. 2025 Feb 27;16:1483794. doi: 10.3389/fmicb.2025.1483794 (PMC11905530; doi:10.3389/fmicb.2025.1483794)

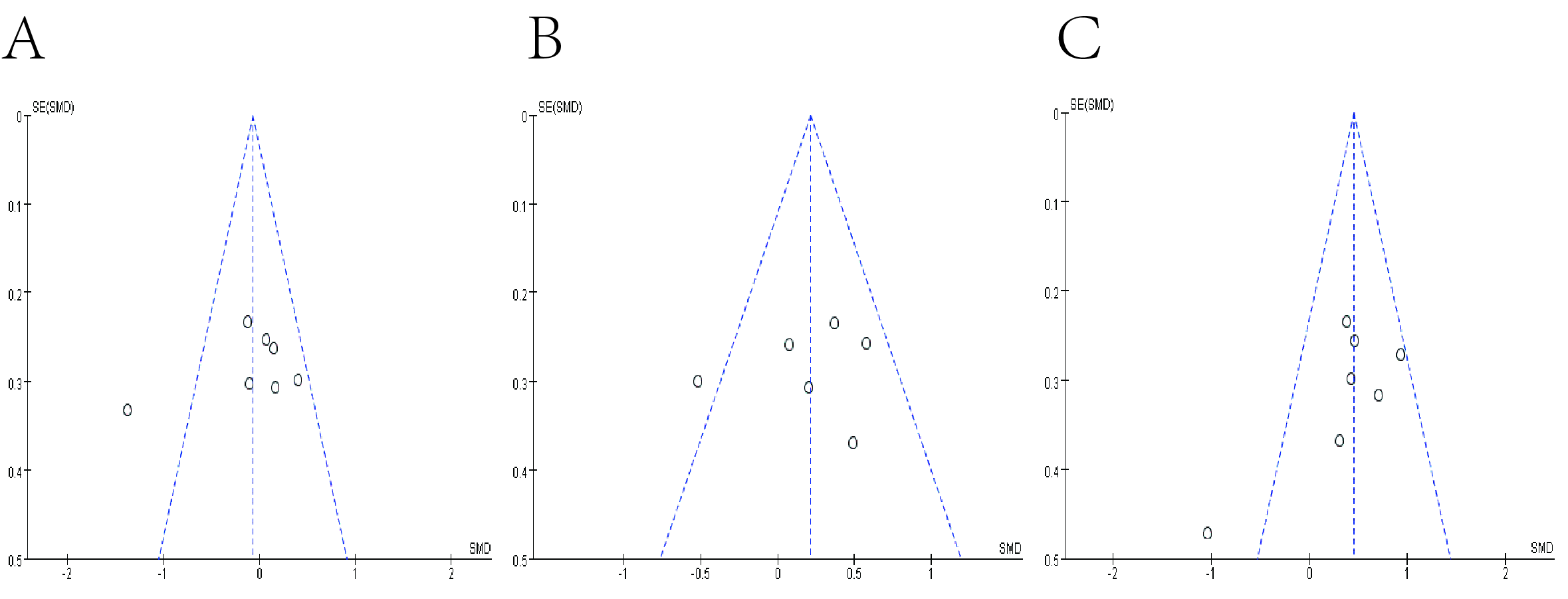

Supplement: Supplementary Figure S1 — Funnel plot of no studies excluded. (A) Bacteroidetes, (B) Blautia, and (C) Bifidobacterium. [file Supplementary_file_1.zip › data sheet 1/Supplementary Figure 1.TIF]

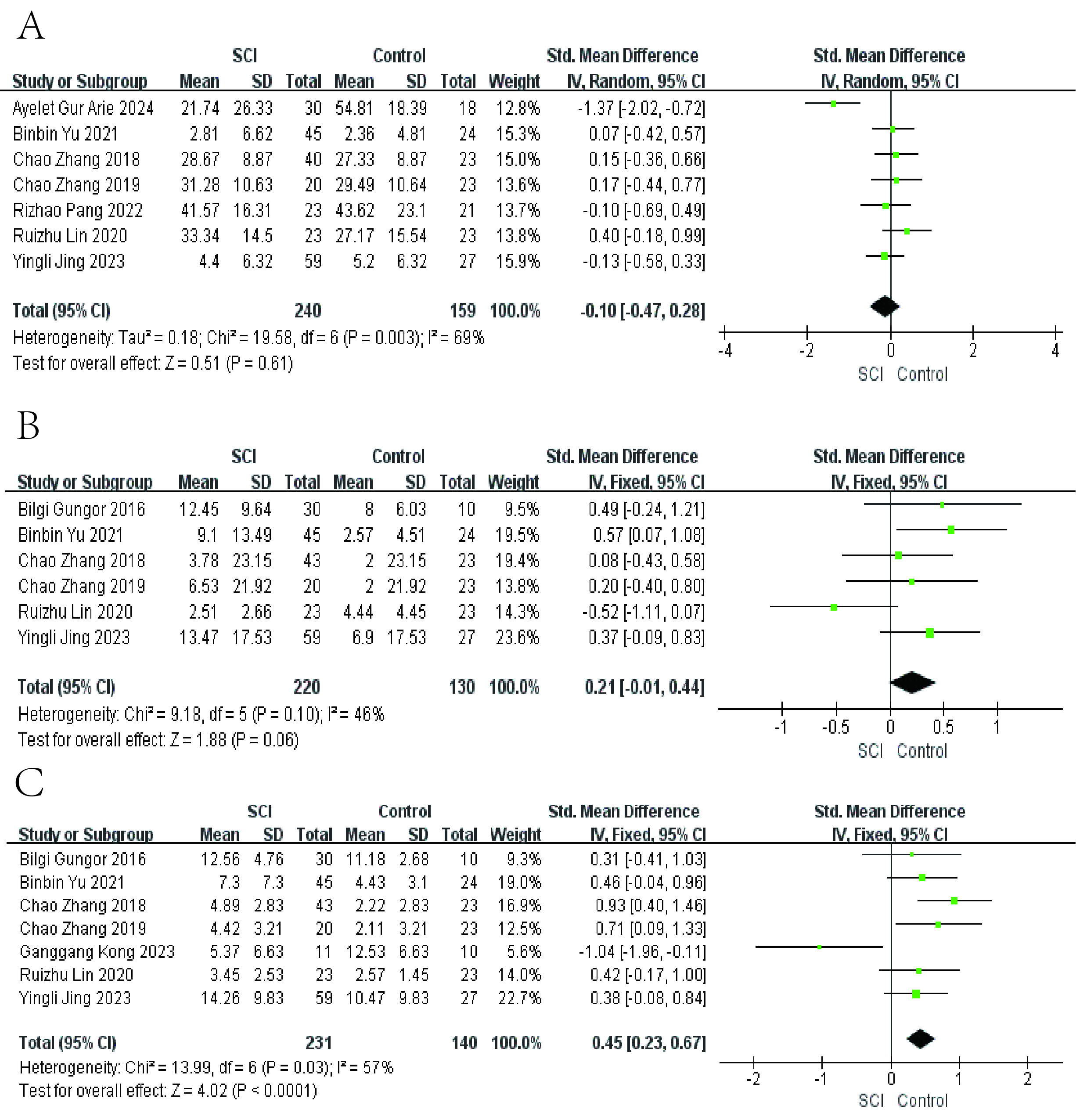

Supplement: Supplementary Figure S1 — Funnel plot of no studies excluded. (A) Bacteroidetes, (B) Blautia, and (C) Bifidobacterium. [file Supplementary_file_1.zip › data sheet 1/Supplementary Figure 2.TIF]

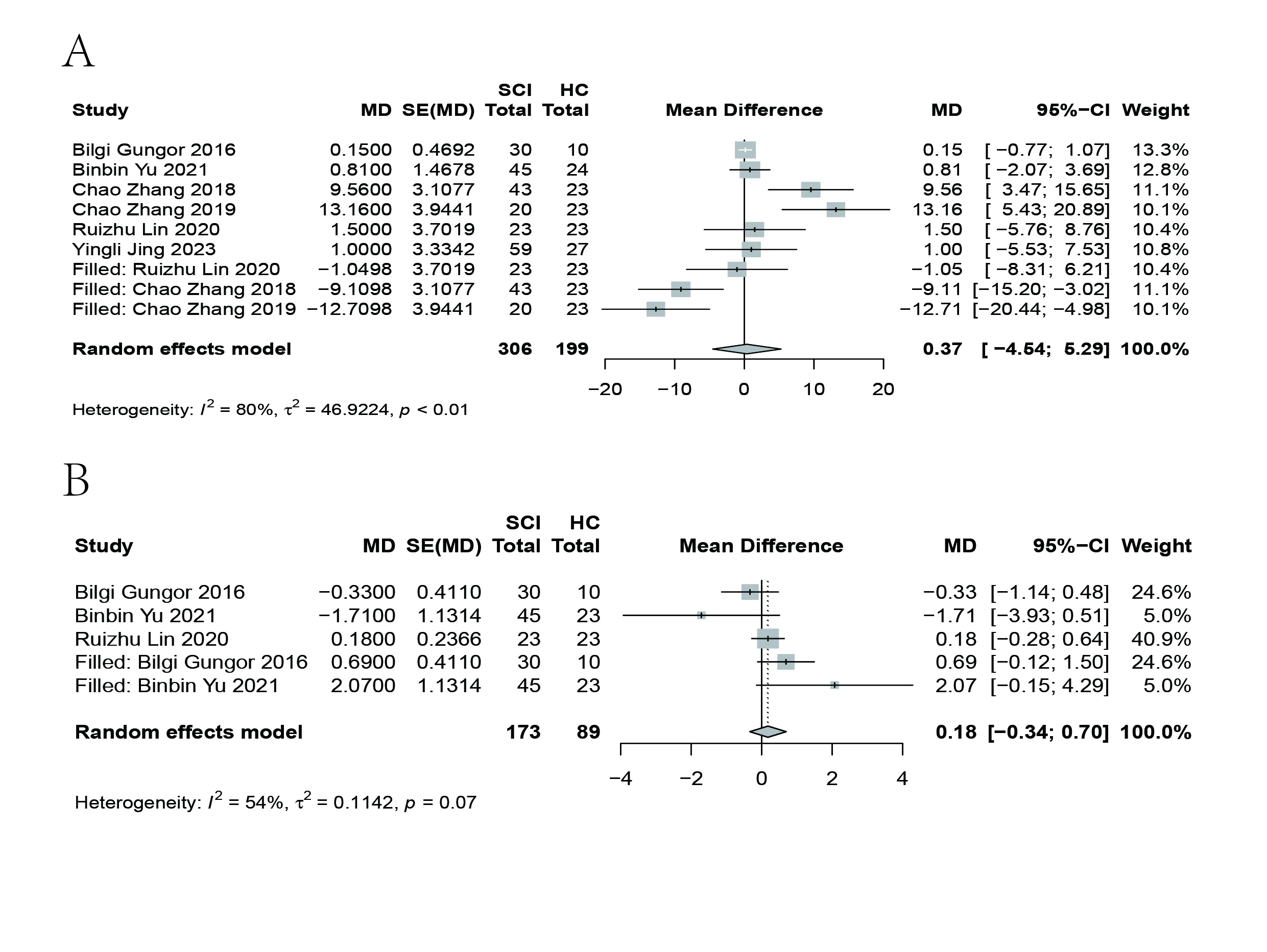

Supplement: Supplementary Figure S1 — Funnel plot of no studies excluded. (A) Bacteroidetes, (B) Blautia, and (C) Bifidobacterium. [file Supplementary_file_1.zip › data sheet 1/Supplementary Figure 3.TIF]
